# More Bone with Less Minerals? The Effects of Dietary Phosphorus on the Post-Cranial Skeleton in Zebrafish

**DOI:** 10.3390/ijms21155429

**Published:** 2020-07-30

**Authors:** Silvia Cotti, Ann Huysseune, Wolfgang Koppe, Martin Rücklin, Federica Marone, Eva M. Wölfel, Imke A. K. Fiedler, Björn Busse, Antonella Forlino, P. Eckhard Witten

**Affiliations:** 1 Evolutionary Developmental Biology Group, Department of Biology, Ghent University, 9000 Ghent, Belgium; silvia.cotti@ugent.be (S.C.); ann.huysseune@ugent.be (A.H.); 2 Department of Molecular Medicine, Biochemistry Unit, University of Pavia, Pavia, 27100 Pavia, Italy; aforlino@unipv.it; 3 SimplyFish AS, 4011 Stavanger, Norway; wolfgang.koppe@simplyfish.no; 4 Department of Vertebrate Evolution, Development and Ecology, Naturalis Biodiversity Center, 2333 Leiden, The Netherlands; martin.rucklin@naturalis.nl; 5 X-ray Tomography Group, Swiss Light Source, Paul Scherrer Institut, 5232 Villigen, Switzerland; federica.marone@psi.ch; 6 Department of Osteology and Biomechanics, University Medical Center Hamburg-Eppendorf, 22529 Hamburg, Germany; e.woelfel@uke.de (E.M.W.); i.fiedler@uke.de (I.A.K.F.); b.busse@uke.uni-hamburg.de (B.B.)

**Keywords:** mineralisation, bone formation, vertebral column, vertebral body fusion, collagen

## Abstract

Dietary phosphorus (P) is essential for bone mineralisation in vertebrates. P deficiency can cause growth retardation, osteomalacia and bone deformities, both in teleosts and in mammals. Conversely, excess P supply can trigger soft tissue calcification and bone hypermineralisation. This study uses a wide range of complementary techniques (X-rays, histology, TEM, synchrotron X-ray tomographic microscopy, nanoindentation) to describe in detail the effects of dietary P on the zebrafish skeleton, after two months of administering three different diets: 0.5% (low P, LP), 1.0% (regular P, RP), and 1.5% (high P, HP) total P content. LP zebrafish display growth retardation and hypomineralised bones, albeit without deformities. LP zebrafish increase production of non-mineralised bone matrix, and osteoblasts have enlarged endoplasmic reticulum cisternae, indicative for increased collagen synthesis. The HP diet promotes growth, high mineralisation, and stiffness but causes vertebral centra fusions. Structure and arrangement of bone matrix collagen fibres are not influenced by dietary P in all three groups. In conclusion, low dietary P content stimulates the formation of non-mineralised bone without inducing malformations. This indicates that bone formation and mineralisation are uncoupled. In contrast, high dietary P content promotes mineralisation and vertebral body fusions. This new zebrafish model is a useful tool to understand the mechanisms underlying osteomalacia and abnormal mineralisation, due to underlying variations in dietary P levels.

## 1. Introduction

Phosphorus (P) is an essential element for a wide variety of biological processes. It plays a key role in cellular metabolism, cell signalling, and the composition of phospholipid membranes and nucleic acids. For all vertebrates, P is crucial for mineralisation of the skeleton, bone, dentin, enamel/enameloid and mineralised cartilage. Naturally our ideas about bone mineral metabolism are influenced by insights that we have obtained from the mammalian (human) model. Vertebrates must control plasma calcium (Ca) within very narrow limits and mammals involve their bone to maintain plasma Ca levels. Thus, Ca deficiency in mammals can have dramatic consequences for the skeleton. This is different for teleosts and other primary aquatic gnathostomes which are able to effectively obtain Ca from the water via the gills (reviewed by [1,2]). When the mineralised skeleton evolved, Palaeozoic aquatic ecosystems were rich in Ca with P as a limiting factor. It thus has been proposed that the early function of bone must have been the storage of P and not the storage of Ca as it is the case in mammals [1,3,4,5].

Bone consists of an organic matrix, mainly represented by fibrillar collagen type I, and a mineral phase composed of Ca and P ions combined in apatite crystals. Osteoblasts, the bone forming cells, secrete non-mineralised collagen matrix known as osteoid, that subsequently mineralises upon the removal of pyrophosphate by alkaline phosphatase, an enzyme produced by osteoblasts [6]. In human bone, osteoid mineralisation may start as late as 10 days after bone matrix formation [7]. Bone formation and mineralisation depends on osteoblasts, whose activity in turn is regulated by osteocytes [8].

Ca and P are closely involved in the development and maintenance of the skeletal system and their adequate intake is crucial to ensure bone health in all vertebrates. Teleost fish can obtain Ca by dietary or gill intake. Only a minimal amount of P can be obtained through the gills, thus like in tetrapods, P remains an indispensable component of the teleost diet [9,10]. Similar to mammals, P homeostasis in teleosts relies on P absorption in the gut, excretion and reabsorption in the kidney and storage in the skeleton [11]. 

P deficiency can occur when dietary P supply is scarce. Dietary P deficiency in Atlantic salmon (*Salmo salar*) causes arrest of bone mineral deposition with no effects on bone matrix secretion [10,12]. Similar effects were described for Nile Tilapia (*Oreochromis niloticus*) [13]. Likewise, P deprivation in murine models causes reduced mineralisation without affecting the production of the organic bone matrix [14,15]. In humans, insufficient dietary P intake is rare [16] and causes hypophosphatemia, hypomineralised bones (osteomalacia) and rickets [15].

In mammals, P insufficiency is thought to be a primary cause of skeletal malformations. Early studies reported that patients suffering from hypophosphatemia are characterised by short stature, bowing of long bones and deformed vertebral column [17,18,19]. Likewise, in teleosts under farming conditions, reduced growth, vertebral column and jaw deformities are related to dietary P deficiency [20,21,22,23,24]. Interestingly, studies on Atlantic salmon under tightly controlled experimental conditions (avoidance of stress, no handling, no vaccination, control of all environmental parameters) with dietary P as a single variable do not show a direct relationship between dietary P deficiency and vertebral column malformations. Animals in their early seawater phase subjected to 10 weeks or 17 weeks of severe dietary P deficiency developed osteomalacia but none of the above mentioned malformations [10,12].

Excess dietary P leads to high serum P concentrations, potentially associated with toxic effects [25,26]. For example, excess dietary P administration may induce metastatic deposition of calcium-phosphate [16]. Humans with normal kidney function but excess dietary P intake develop abnormal vascular calcification [27,28,29]. Studies on dogs that were fed a high P diet demonstrated increased soft tissue calcification, particularly in the kidney [30,31,32,33] and increased accretion of cortical bone [34].

A better understanding of the effects caused by scarce and excess dietary P conditions on the skeleton can expand the current knowledge on the mechanisms underlying osteomalacia and abnormal mineralisation and provide better insight into the mineralisation process. *Danio rerio* (zebrafish) is an established model for the study of bone formation, given that basic bone cell differentiation pathways and ossification processes have been conserved across vertebrates [35]. This study examines the post-cranial skeleton in zebrafish, given that the vertebral column is the most studied anatomical structure in biomedical research and in aquaculture [36,37]. Likewise, fin rays are popular for the study of bone regeneration and fish health [38,39]. Focusing on both endoskeletal and dermal skeletal elements, this study shows in detail how structure and mineralisation of different bone structures are affected by dietary P content. With this new zebrafish model it is possible to falsify the following hypotheses: (a) under low P conditions, bone mineralisation stops but bone matrix formation increases without affecting the morphology of the bones and without causing vertebral body malformations; (b) high dietary P levels increase bone mineralisation, bone stiffness and promote vertebral body fusions; (c) bone matrix secretion and mineralisation are uncoupled processes at the level of cellular and subcellular level. This new zebrafish model represents a valuable tool to elucidate the primary effects of low and high dietary P levels on bone formation and mineralisation. 

## 2. Results

### 2.1. Zebrafish Growth Depends on Dietary P

**Figure 1:**
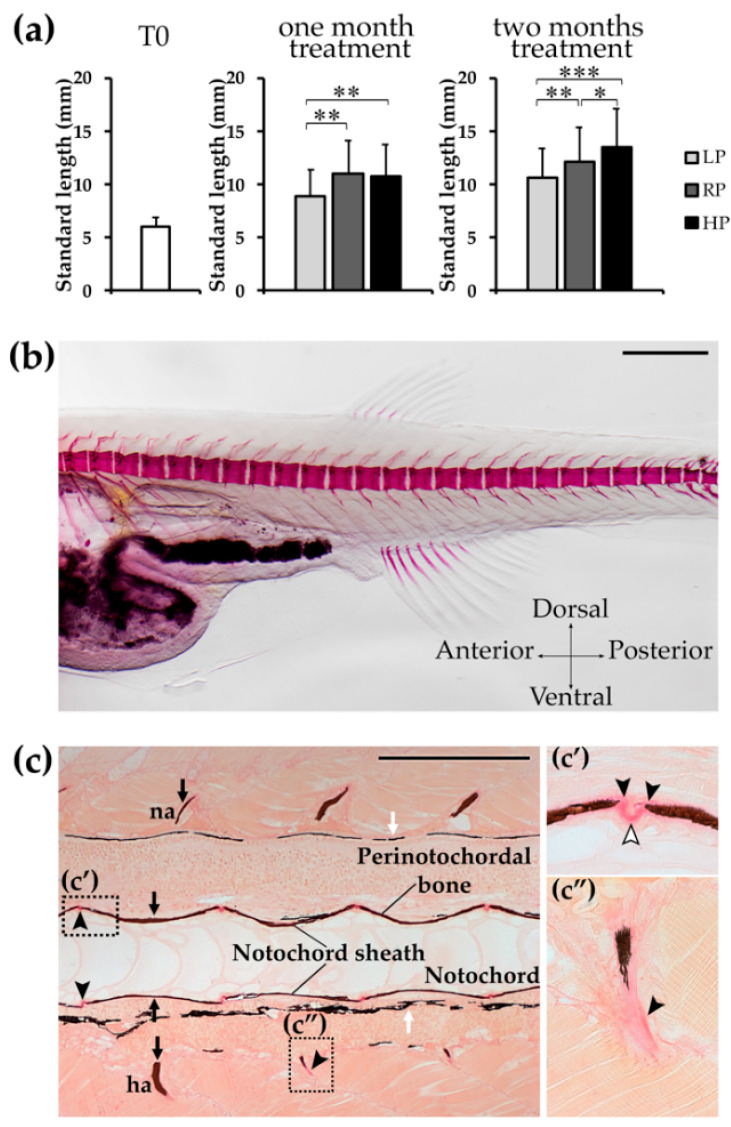
Zebrafish growth and T0 animals. (**a**) Morphometric analysis of WT zebrafish at the beginning of the experiment (T0, 28dpf) and after one and two months of treatment with the experimental diets. Animals treated with the low P diet (LP) are smaller than controls (RP) and high P diet (HP) treated animals, whereas HP zebrafish present a significantly increased standard length. Mann-Whitney test, *: *p* < 0.05; **: *p* < 0.01; ***: *p* < 0.001. (**b**) T0 zebrafish, prior the beginning of the experiment, stained with Alizarin red S shows normally developed vertebral column and forming vertebral bodies. No vertebral column malformations, nor vertebral body fusion or compression are present. Scale bar: 500 μm. (**c**) Notochord sheath, perinotochordal membranous bone and neural (na) and haemal (ha) arches are mineralised in T0 animals, as shown by Von Kossa/Van Gieson staining on sagittal sections. Vertebral bodies are normally shaped and spaced. High magnification panels show (**c’**) vertebral endplates with osteoid (black arrowheads) and intervertebral ligament (white arrowhead, see Figure 6a for details), (**c”**) haemal arch with non-mineralised collagen matrix (black arrowhead). Mineralised bone: brown (black arrows); pigment: black (white arrows), non-mineralised collagen matrix/osteoid: red (black arrowhead). Scale bar: 200 µm.

A comparison of the standard length (SL) reveals that zebrafish treated with the low P (LP) diet are significantly smaller compared to the controls (regular P diet, RP) and high P (HP) diet treated animals after one month of treatment (Figure 1a and Table 1). This difference becomes more evident after two months of treatment. In contrast, HP animals have significantly increased SL compared to LP at both timepoints and compared to RP after two months of treatment.

### 2.2. T0 Animals Display a Mineralised Vertebral Column without Any Anomaly

At the beginning of the experiment (T0), control specimens were analysed by Alizarin red S whole mount staining to establish the degree of vertebral column mineralisation and possible malformations. Bird and Mabee [40] serve as reference for normal skeletal development in zebrafish. In addition several publications define malformations of the zebrafish axial skeleton [41,42,43]. The absence of any of the described malformation is regarded as normal in this study. None of the analysed samples presented malformations such as vertebral centra fusion, vertebral centra compression, curled or supernumerary arches (Figure 1b). Histological analysis of sagittal sections further confirmed that vertebral bodies do not show malformations and that the notochord sheath, perinotochordal membrane bone, and arches are properly mineralised (Figure 1c). 

### 2.3. Mineralisation of Endoskeleton and Dermal Skeleton Is Arrested under Low P Conditions

**Figure 2:**
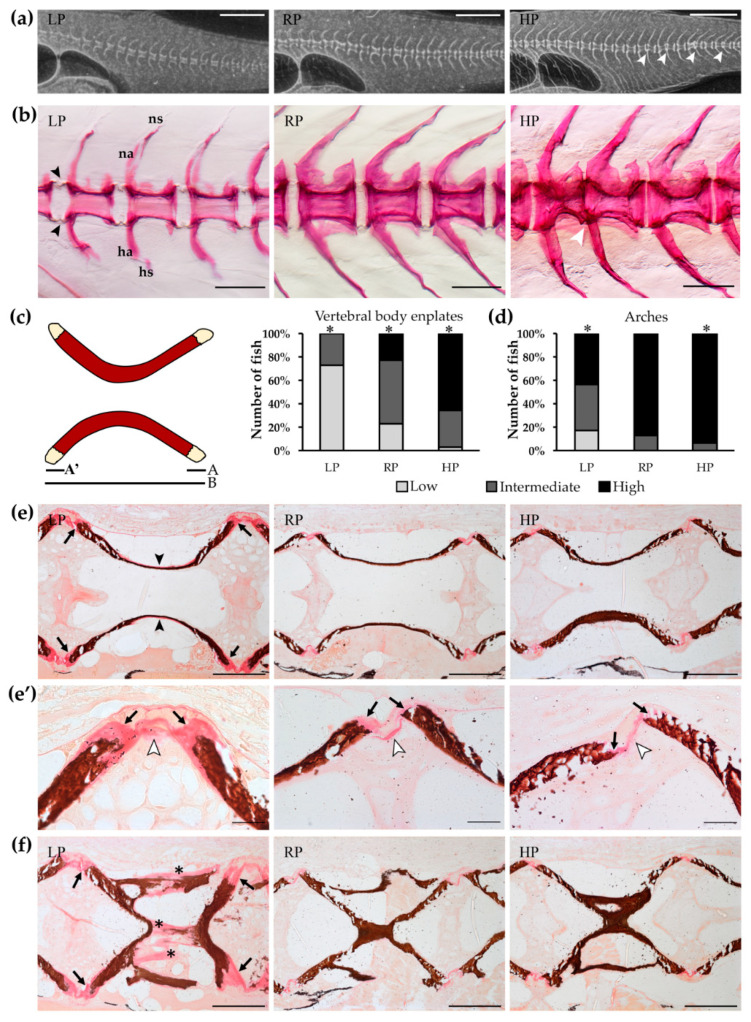
Mineralisation levels of vertebral column after two months of dietary treatment. (**a**) Vertebral column of low P diet (LP) treated animals is characterised by reduced radiodensity compared to controls (RP) and high P diet (HP) treated animals. HP zebrafish present multiple vertebral body fusions (white arrowheads). Scale bar: 1 mm. (**b**) Alizarin red S staining of vertebral columns shows vertebral body endplates (black arrowheads) characterised by low mineralisation levels in LP animals, intermediate mineralisation in controls and high mineralisation levels in HP animals. HP zebrafish present vertebral body fusion (white arrowhead). Neural (na) and haemal arches (ha) are low-mineralised and their spines (ns, hs, respectively) are deformed in LP individuals compared to RP and HP zebrafish. Scale bar: 200 µm. (**c**) Quantitative analysis of vertebral body endplate mineralisation: the non-mineralised endplate is expressed as percentage of the total non-mineralised endplate length (A + A’) over the total vertebral length (B), (A + A’)/B. Chi squared or Fisher’s exact test, pairwise comparison, *: *p* < 0.05. (**d**) Qualitative analysis of arch mineralisation levels. Please see Materials and Methods for further details. Chi squared or Fisher’s exact test, pairwise comparison, *: *p* < 0.05. (**e**) Sagittal sections of vertebral bodies show large areas of non-mineralised matrix at the level of the vertebral endplates (black arrows) in LP animals compared to RP and HP animals. LP individuals present also a thin osteoid layer in the outer part of the vertebral body (black arrowheads), completely absent in RP and HP animals. Scale bar: 100 µm. (**e’**) High magnification of endplates in panel **e**. Extended non-mineralised bone matrix (black arrows) is visible in the vertebral endplates of LP animals. RP and HP zebrafish present thin endplates with reduced osteoid (black arrows). White arrowheads indicate intervertebral ligaments (see Figure 6a for details). Scale bar: 20 µm. (**f**) Mineralisation of bone trabeculae (asterisks) is also affected by low P levels in the diet. Black arrows: endplates. Von Kossa/Van Gieson staining: mineralised bone: brown; non-mineralised collagen matrix/osteoid: red. Scale bar: 100 µm.

After two months of treatment, X-rays of vertebral columns from LP individuals show reduced radiodensity compared to vertebral columns of RP and HP animals (Figure 2a). To better investigate the LP phenotype, bone mineralisation levels were evaluated on whole mount Alizarin red S stained specimens. In comparison to the control group (RP diet), LP animals display an overall low-mineralised endoskeleton, including vertebral body centra and neural and haemal arches and spines. After one and two months under low P conditions, the majority of the animals have non-mineralised vertebral body endplates and largely non-mineralised neural and haemal arches. Conversely, the HP diet shows enhanced mineralisation of vertebral body endplates, neural and haemal arches. These structures are high mineralised after one and two months of dietary treatment in all the HP animals analysed (Figure 2b–e, Supplementary Table S1). Compared to controls, LP and HP fish do not show a completely homogenous phenotype after one month of dietary treatment: a small percentage (8%, four fish out of 51) of LP animals shows fully mineralised vertebral body endplates and some HP individuals (19%, six fish out of 31) present non-mineralised endplates. However, all HP animals have fully mineralised vertebral body endplates after two months of HP diet (Supplementary Table S1). The histological analysis on non-demineralised sagittal sections of the vertebral column confirms these findings. Von Kossa staining for P allows the precise distinction between mineralised and non-mineralised bone (osteoid), comparable to the whole mount Alizarin red S staining for Ca. Both techniques show large amounts of non-mineralised collagen matrix at the rim of vertebral body endplates in LP animals. Moreover, sections stained with Von Kossa show that LP vertebral bodies are surrounded by large amounts of non-mineralised bone matrix, and vertebral centra bone trabeculae and arches present a similar phenotype (Figure 2f). In contrast, vertebral body endplates and arches in control animals (RP diet) have narrow osteoid layers, indicative for fast mineralisation. The HP animals display an extremely thin osteoid layer at the vertebral endplates and fully mineralised arches (Figure 2e). The extent of bone matrix mineralisation level coincides with the dietary P content (Supplementary Table S1).

**Figure 3:**
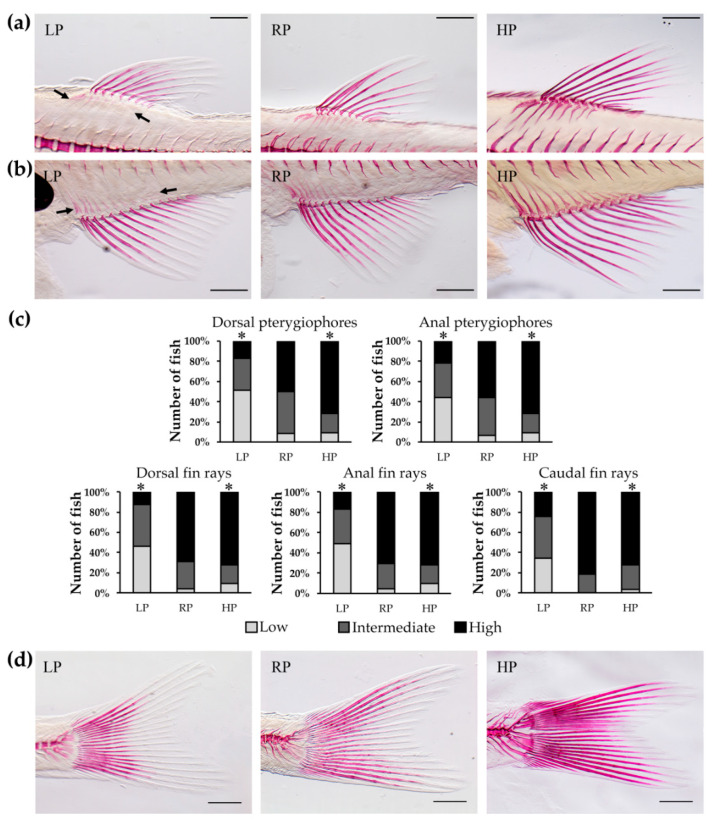
Mineralisation levels of median fin structures after two months of dietary treatment. Pterygiophores or radials, endoskeletal structures supporting the dorsal (**a**) and anal (**b**) fins (black arrows), present reduced mineralisation levels in low P diet (LP) treated zebrafish, in comparison with controls (RP) and high P diet (HP) fed animals. Likewise, Alizarin red S staining shows impaired mineralisation of dorsal and anal fin rays in LP animals. Scale bar: 500 µm. (**c**) Analysis of pterygiophores and quantitative analysis of fin rays mineralisation levels. Pterygiophores mineralisation levels were qualitatively evaluated as low, intermediate or high depending on Alizarin red S distribution in the bone. Fin rays mineralisation levels were quantitatively analysed: low, more than two non-mineralised segments; intermediate, one or two non-mineralised segments; high: all segments mineralised. Chi squared or Fisher’s exact test, pairwise comparison, *: *p* < 0.05. (**d**) Caudal fin rays stained with Alizarin red S display reduced mineralisation in LP animals compared to RP and HP zebrafish. Scale bar: 500 µm.

Similar to vertebrae, mineralisation of the fin endoskeletal support elements (pterygiophores or radials) [40] is affected after one and two months by dietary P. LP animals display a low or intermediate extent of bone matrix mineralisation, RP animals display intermediate mineralisation levels and HP animals have fully mineralised radials (Figure 3 and Supplementary Table S1).

Dietary P affects also the mineralisation of the dermal skeleton as evident from the analysis of the lepidotrichia, which are paired fin ray segments that mineralise [44]. Depending on dietary P content, lepidotrichia show progressively increasing mineralisation levels in the dorsal, anal, and caudal fin. After two months of treatment, the LP animals show low or intermediate lepidotrichia mineralisation. In contrast, in RP and HP animals the fin ray segments are fully mineralised (Figure 3 and Supplementary Table S2).

### 2.4. Dietary P Has No Effect on Vertebral Morphology but HP Animals Have Fused Vertebral Centra

Detection of vertebral column abnormalities was performed on whole mount Alizarin red S stained specimens. After one or two months of treatment, none of the dietary groups display bending of the vertebral column (kyphosis, scoliosis, or lordosis). Vertebral bodies present in general a normal shape and size (but see below, vertebral fusion in HP animals). On the contrary, deformities of neural and haemal spines are present in most LP animals (Figure 2b), suggesting that the non-mineralised collagen matrix is easily deformable by muscle contraction.

No deformities of vertebral body centra occur in LP and RP animals except a few cases of vertebral fusion and compression at both analysed timepoints (Table 2). Zebrafish treated for two months with the HP diet present an increased frequency of vertebral body fusions. More than a quarter, 28%, of the analysed specimens present at least one vertebral centra fusion. The increased occurrence of fusions in HP zebrafish is statistically significant (*p* = 0.006). Interestingly, six out of nine animals that suffer from vertebral fusion have multiple fusions in the caudal region [40] (Table 2 and Figure 2a). This suggests that high dietary P supply might promote vertebral body fusions.

### 2.5. High Dietary P Is Associated with a Higher Stiffness in the Vertebral Endplates

**Figure 4:**
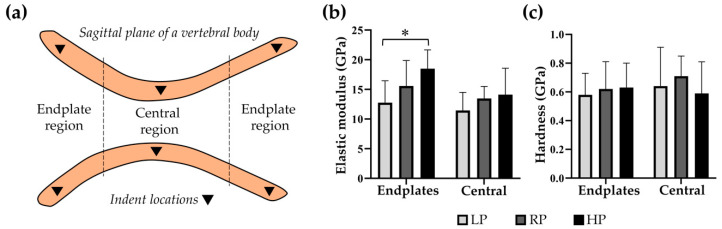
High dietary P is associated with higher stiffness in the bone formed after two months of treatment. (**a**) Schematic representation of nanoindentation measurements in the sagittal plane of a vertebral body. (**b**) In the endplates, the elastic modulus as indicator for stiffness shows significantly higher values in HP compared to LP (*: *p* = 0.004), and a trend towards higher values in HP compared to RP (*p* = 0.073). In the central region, all dietary groups showed similar values. (**c**) The hardness of the vertebrae was similar in all dietary groups in both the endplates and central regions.

To assess if changes in dietary P influence the mechanical properties of the vertebral tissue, nanoindentation was performed (Figure 4a). In the vertebral endplate regions, a significantly higher elastic modulus is noted in HP zebrafish with 18.48 ± 3.18 GPa compared to LP zebrafish with 12.77 ± 3.68 GPa (*p* = 0.004), and a trend towards higher elastic modulus in HP compared to RP zebrafish with 15.57 ± 4.30 GPa (*p* = 0.073). In the central region, the elastic modulus is similar in all dietary groups with 11.44 ± 3.05 GPa in LP, 13.47 ± 2.01 GPa in RP, and 14.11 ± 4.47 GPa in HP zebrafish (Figure 4b). Endplate hardness values are different with 0.58 ± 0.15 GPa in LP, 0.62 ± 0.19 GPa in RP, and 0.63 ± 0.17 GPa in HP zebrafish, but for the extremely small probes it was not possible to establish statistical significance. The same applies for hardness values in the central region, with 0.64 ± 0.27 GPa in LP, 0.71 ± 0.14 GPa in RP, and 0.59 ± 0.22 GPa in HP zebrafish (Figure 4c).

### 2.6. LP Individuals Have Increased Bone Matrix Formation and Highly Active Osteoblasts

**Figure 5:**
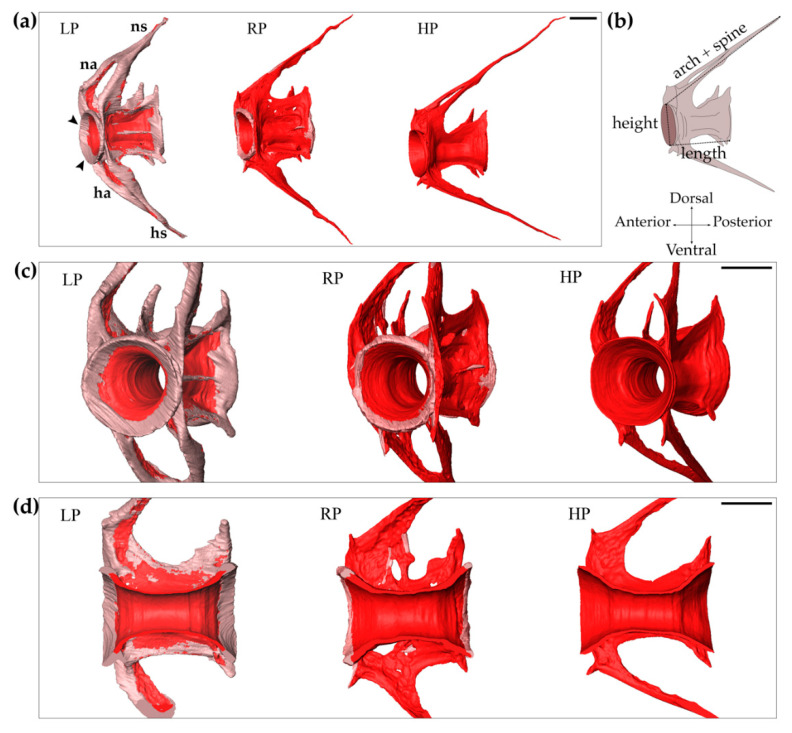
Increased bone formation in LP zebrafish after two months of dietary treatment. Synchrotron X-ray tomographic microscopy scans reveal an increased amount of non-mineralised matrix in the vertebral body and arches of low P diet (LP) treated animals compared to controls (RP) and high P diet (HP) treated individuals. (**a**) Lateral view of the 10th caudal vertebral body, neural (na) and haemal (ha) arches and their spines (ns, hs, respectively). Vertebral endplates are indicated by black arrowheads. Scale bar: 100 µm. (**b**) Schematic representation of the measured parameters: vertebral body length, vertebral body height, length of arch plus spine. (**c**) Frontal view of vertebrae. Scale bar: 100 µm. (**d**) Virtual sagittal sections of the vertebral bodies. Scale bar: 100 µm. Non-mineralised bone: pink; mineralised bone: red.

Synchrotron based X-ray tomographic microscopy scans of a representative, similar-sized, specimen from each of the dietary groups reveal microstructural differences in the vertebrae of treated animals which are difficult to assess on whole mount Alizarin red S stained specimens. Although the analysed vertebral bodies in the caudal region [40] of the vertebral column of LP, RP, and HP individuals have a similar length and height, the length of arches and spines varies, with a maximum value in the HP group and a minimum value in the LP group (Figure 5 and Supplementary Table S3). Instead, the total vertebral body bone volume, calculated as the volume of vertebral body centra plus haemal and neural arches and haemal and neural spines, strongly differs between the representatives of the three diet groups. The total bone volume reaches a maximum in the individual from the LP group and a minimum in the HP animal. The RP individual has an intermediate bone volume. A pronounced increase in non-mineralised bone matrix is observed in the LP specimen compared to the RP and HP animals (Figure 5 and Supplementary Table S3). Extensive non-mineralised bone matrix is localised at both vertebral endplates, at the neural and the haemal arches and at the neural and haemal spines in the LP animal. Volume data confirms that the volume of the newly formed bone in the LP vertebra is larger compared to the HP vertebra. In the individual from the HP group, endplates, arches, and spines are completely mineralised but thinner than in RP vertebral bodies (Figure 2e,f and Figure 5).

**Figure 6:**
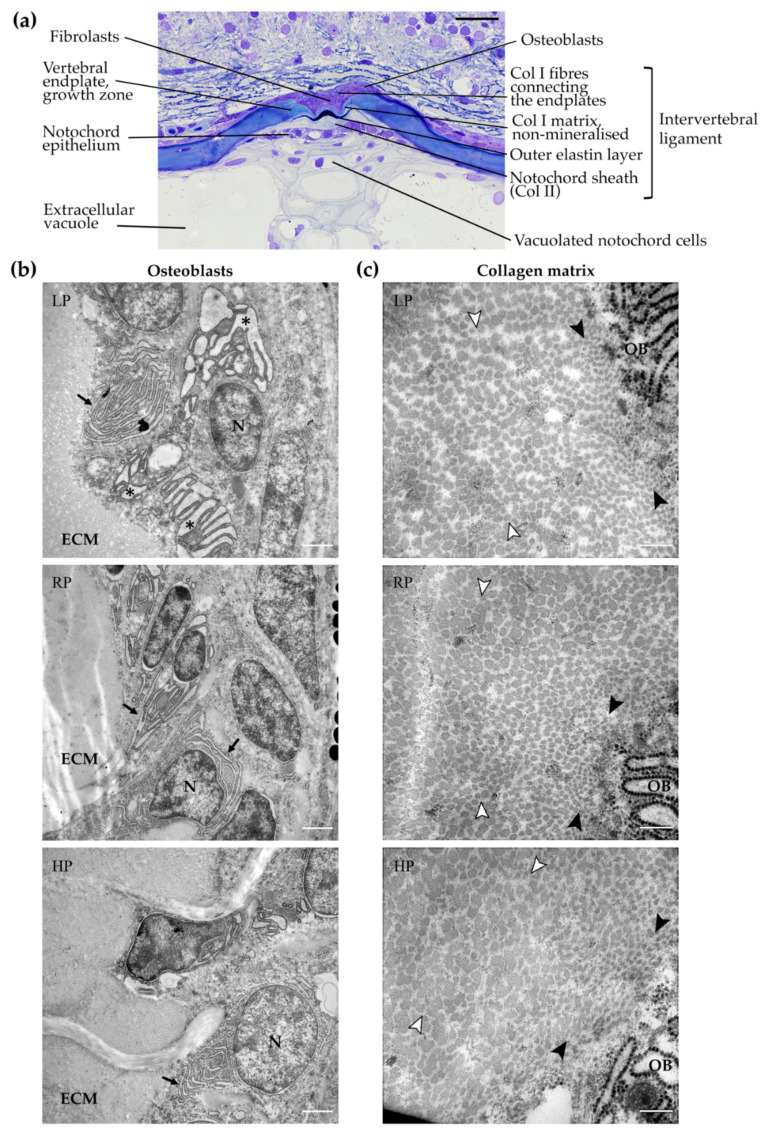
Osteoblasts and collagen type I in the vertebral body endplate growth zone. (**a**) Representative toluidine blue stained semi-thin sagittal section showing internal structures of zebrafish vertebral centra and intervertebral ligament. Vertebral endplates are normally spaced and fully extended in all dietary groups. The notochord sheath is composed of collagen type II (Col II) secreted by the cells of the notochord epithelium, also named chordoblasts. Vertebral bodies are interconnected by the notochord sheath and by collagen type I (Col I) fibres outside the notochord. All structures of the intervertebral ligament are unaltered. Osteoblasts in the vertebral endplate growth zone are located outside the notochord sheath between collagen type I fibres. Inside, the notochord is composed by vacuolated notochord cells and extracellular vacuoles. Scale bar: 20 µm. (**b**) Transmission electron microscopy images of osteoblasts in the vertebral endplate growth zone after two months of dietary treatment. Osteoblasts are active and present a high number of endoplasmic reticulum (ER) cisternae (black arrows), which are enlarged in low P diet (LP) treated animals (asterisks) compared to controls (RP) and high P diet (HP) treated animals, indicative of increased bone matrix production. ECM: extracellular matrix, N: nucleus. Scale bar: 1 µm. (**c**) Higher magnification of collagenous bone matrix located at the vertebral endplates. Collagen type I fibres in the immediate vicinity of osteoblasts (OB) have similar diameters among the three dietary groups, as well as collagen fibres located at a distance from the osteoblasts, within the extracellular matrix, indicative of unaltered fibre maturation. Black arrowheads: fibres in the vicinity of the osteoblasts with small diameters; white arrowheads: fibres at a distance from osteoblasts with large diameters. Scale bars: 200 nm.

Histological analysis showed that, in all dietary groups, intervertebral spaces are unaltered with normal intervertebral ligaments. The vertebral endplates are fully elongated without any malformation. Numerous osteoblasts are present in the growth zone of the vertebral body endplates (Figure 6a). Transmission electron microscopy of representative specimens confirmed the presence of active osteoblasts in all dietary groups. All osteoblasts are characterised by a high number of endoplasmic reticulum (ER) cisternae. In the osteoblasts of the LP individual, ER cisternae are enlarged compared to the RP and HP individuals, indicative of increased cellular activity (Figure 6b).

### 2.7. Collagen Type I is Unaltered in All Dietary Groups

Electron microscopy was used to analyse collagen type I fibres in the bone growth zone of the vertebral endplates. Newly secreted collagen fibres in the close proximity of osteoblasts have similar diameters in animals of all three dietary groups (LP: 25.2 ± 5.4 nm; RP: 26.3 ± 7.3 nm; HP: 25.9 ± 5.3 nm). Likewise, matured collagen fibres in the osteoid at a distance from the osteoblasts have similar diameters (LP: 49.1 ± 11.0 nm; RP: 53.1 ± 10.8 nm; HP: 52.6 ± 9.4 nm) (Figure 6c).

**Figure 7:**
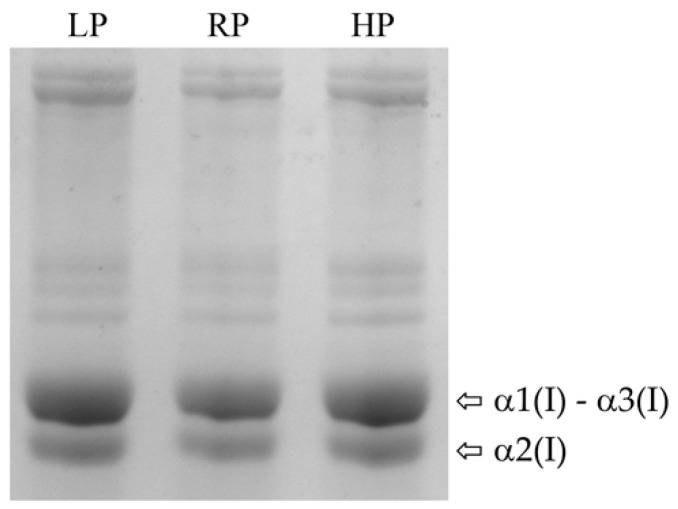
Electrophoretic analysis of collagen type I. Coomassie stained SDS-Urea-PAGE of collagen type I extracted from bone of low P diet (LP) treated zebrafish, controls (RP) and high P diet (HP) treated animals (pool of 2 samples per dietary group). Zebrafish present three collagen type I α chains (α(I)), named α1(I), α3(I) and α2(I). Collagen α(I) chains show bands with similar electrophoretic migration in all dietary groups.

Given that activity of ER resident enzymes involved in collagen post-translational modification does not depend on P [45], we hypothesised that normal collagen post-translational modification occurs in all dietary groups. SDS-Urea-PAGE analysis of collagen type I pepsin-extracted from bone shows similar electrophoretic migration of the α(I) chains bands in LP, RP, and HP animals, suggesting normal collagen post-translational modifications in all dietary groups [45] (Figure 7).

## 3. Discussion

This study describes the primary effects of low and high dietary phosphorus (P) content in juvenile zebrafish. P is a critical element for several biological processes, including hard tissue mineralisation. An extended period of dietary treatment with different P levels affects growth, bone formation, bone mineralisation and bone mechanical properties. In particular, low dietary P (LP) level causes growth retardation but also increases non-mineralised bone matrix production, as indicated by histology and synchrotron X-ray tomographic microscopy. Endoplasmic reticulum cisternae of osteoblasts are enlarged, indicative for increased collagen synthesis, in line with the observed increase of bone matrix production. Conversely, high dietary P (HP) level increases growth, bone mineralisation and stiffness and promotes vertebral body fusions. Collagen post-translational modification, structure and arrangement of collagen fibres in the bone matrix are not influenced by dietary P content.

### 3.1. Skeletal Mineralisation Arrest under Low P Conditions

The arrest of bone matrix mineralisation without the stop of bone matrix production is the primary effect of the LP diet on the skeleton of juvenile zebrafish. Structure and shape of the non-mineralised bone are normal. It can thus be defined as bone according to De Ricqlès et al. (1982): “mineralisation in bone can be missing alone or in combination with other components. Nevertheless, for reasons dealing with composition, homology, origin and function the tissue should be recognised as bone” [46]. The LP zebrafish model recapitulates the bone phenotype typical of P deficiency that is also observed in other vertebrates. Indeed, mammals, including humans and rats, and teleost fish under dietary P deficiency, show bones with reduced radiodensity and an increased amount of osteoid at the level of epiphyseal plates [15], endosteal bone [14], pharyngeal bone [13], and vertebral centra [10]. Staining for mineral detection (whole mount Alizarin red S for Ca, Von Kossa on sections for P) reveals the lack of bone mineralisation in LP animals. Endoskeletal elements, such as vertebral body centra, haemal and neural arches, and elements of the dermal skeleton, such as fin rays, are equally affected (Figure 2 and Figure 3, Supplementary Tables S1 and S2). Given that growing juvenile zebrafish were exposed for two months to low dietary P content, the presence of hypomineralised bones was expected. Still, bones are not only hypomineralised, but new bone matrix is formed and this matrix has no minerals. Our findings match previous studies that analysed the consequences of dietary P deficiency in Atlantic salmon (*Salmo salar*) [10,12,20] and in Nile Tilapia (*Oreochromis niloticus*) [13]. Moreover, the observed phenotype resembles the bone phenotype detected in murine models of heritable hypophosphatemia [47,48,49], a disorder related to low P levels in the blood [19,50]. Likewise, patients suffering from rickets present hypomineralised bones [15].

Conversely, in animals in this study that received a diet with regular P content (RP), bone mineralisation was normal and in line with previous studies that traced zebrafish skeletal mineralisation [40]. All bone structures analysed in the RP group present a small amount of non-mineralised bone identifiable as osteoid. Bone elements from HP fish are even further mineralised, the osteoid is extremely narrow.

This study shows that a low P diet arrests mineral deposition equally in endo- and dermal skeletal elements. The mineralised endoskeleton evolved much later than the mineralised dermal skeleton [51]. The latter comprises teeth, scales and fin rays [35,52,53]. In the present study, the degree of dermal fin ray mineralisation coincides with the degree of vertebral body mineralisation (Figure 2 and Figure 3, Supplementary Tables S1 and S2). Likewise, a zebrafish mutant strain called nob (no bone) that completely lacks bone mineralisation presents non-mineralised dermal and endoskeletal elements to the same extent [54]. That fin rays can serve as indicators to track skeleton mineralisation has immediate applications. It will allow monitoring the mineralisation status of the overall skeleton related to dietary P content in vivo, using vital mineral staining for fin rays [55]. Such a non-invasive method avoids animal sacrifice in the context of low or high dietary P treatment or in other experiments which trace skeletal mineralisation.

### 3.2. A Functional and Unaltered Axial Skeleton Despite Low Dietary P

In teleosts, particularly in farmed salmonids, dietary P deficiency has been linked to vertebral column malformations such as vertebral body compression and fusion [20,21,22,23,24]. In this study, low dietary P content for two months does not cause vertebral centra deformities in juvenile zebrafish. Despite the lack of bone mineralisation, vertebral centra have a normal shape without alterations that would foreshadow vertebral body compression or fusion [56,57]. Different from the unaltered centra, neural and haemal spines are twisted in LP animals. This phenotype has been described as sign of P deficiency in farmed teleost species such as Atlantic salmon, haddock (*Melanogrammus aeglefinus*), and halibut (*Hippoglossus hippoglossus*) [58,59,60]. Similarly, low-mineralised neural and haemal spines in LP zebrafish have an undulated shape (Figure 2b), yet without signs of fracture. Indeed, the collagen-based bone matrix alone is a very tough material. The toughest known vertebrate bones are deer antlers, which can flex without damage due to their low degree of mineralisation [61,62]. Notably, vertebral centra and arches and their spines are developmental modules, meaning that the control of their development is to a large degree independent [63,64,65]. This could explain why spines are twisted but vertebral centra are not affected in LP zebrafish. In laboratory zebrafish strains, undulated spines also occur linked to conditions other than P deficiency such as increased rearing density [43] or disturbed somite formation [66]. In addition, it has been suggested that spine deformities relate to musculature impairment [67,68]. The comparison of the results from this study with other studies that encountered malformed spines can, however, be difficult. Other studies have used different species or different zebrafish strains, different rearing conditions and different diet formulations. Costa et al. [69] tested the effect of six diets with different P levels on the zebrafish skeleton, but the diet composition was different from the one used in the present study. The diets contained poultry visceral meal and soy bean oil, whereas the diet used in this experiment contains krill meal, fish meal, and fish oil. Moreover, the inorganic P source in the present study is monoammonium phosphate (MAP), whereas dicalcium phosphate (DCP) was used by Costa et al. [69]. Solubility and digestibility, and thus the bioavailability of MAP, are considerably higher than DCP [70,71]. This and other dietary ingredients could explain why our LP zebrafish do not develop vertebral centrum deformities, or other deformities encountered by Costa et al. [69], such as severe bending of the vertebral column or craniofacial malformations. The phenotype of the control group (RP) in the present study, 1.0% total P based on MAP supplement, equals the phenotype obtained by Costa et al. [69] with the diet containing 1.85% total P based on DCP supplement.

The absence of vertebral centra malformations in the LP zebrafish group is in line with what is observed in recent studies on Atlantic salmon. In two different experiments, animals in their early seawater phase received P-deficient diets (50% of the total P requirement) for 10 weeks and 17 weeks. Like LP zebrafish in the current study, Atlantic salmon developed bone without minerals but no vertebral column deformities [10,12]. It can of course not be excluded that a prolonged low P period would eventually generate skeletal malformations in growing zebrafish.

How can the absence of malformations in LP zebrafish be explained? From a functional point of view, the notochord alone in the absence of vertebral bodies can act as efficient axial skeleton. This is the case in teleost fish that hatch as embryos [72,73] and in basal adult osteichthyans [74]. Members of several stem-ward groups, which comprise large animals such as dipnoans (lungfishes), coelacanths (crossopterygians) and sturgeons (chondrosteans, up to six meters in length), have a continuous non-constricted notochord as functional axial skeleton and do not develop mineralised vertebral centra [74,75,76]. Also, the nob zebrafish mutant strain that completely lacks bone mineralisation, shows correctly patterned but non-mineralised vertebral body anlagen [54]. Moreover, several species of deep sea fish are characterised by extremely low-mineralised skeletons and low-mineralised vertebral centra [77,78,79]. Thus, a functional and healthy notochord that supports the axial skeleton may compensate for non-mineralised vertebral centra as it compensates for absence in other osteichthyans. Indeed, this study demonstrates that a two months period under low P conditions does not cause any morphological alteration of the vertebral centra in zebrafish, except osteomalacia. Internal vertebral centra structures appear unaltered on histological sections. Intervertebral ligaments and the notochord tissue in the intervertebral space of LP zebrafish (a region called intervertebral disk by Schaeffer [80]) remain intact. This is an important observation because, also in teleosts, vertebral body malformations typically start with alterations of the intervertebral disk [56,57,81]. These findings strengthen the idea that P deficiency alone is not a primary cause of vertebral column abnormalities in zebrafish, and that other or additional factors trigger the development of malformations. Other factors that are currently discussed to cause vertebral column malformation in zebrafish and other teleost species are rearing temperature, excess swimming, increased rearing density or dietary vitamin A supply [42,43,82,83].

### 3.3. Is There a Link between Excess P Content and Vertebral Fusion?

In the present study, zebrafish from the HP group present an increased frequency of vertebral centra fusion. This suggests that high rather than low dietary P content could be a causative factor for vertebral body fusion in zebrafish. Up to now, little is known about the effects of dietary P excess on the development of skeletal malformations in teleosts [59]. In mammals, however, ectopic mineralisation can be caused by excess dietary P intake. Humans with excess dietary P ingestion develop abnormal mineralisation of the vascular tissue [27,28,29]. Dogs treated with high P diets developed mineralisation in the kidney [30,31,32,33] and increased accretion of cortical bone [34]. Also metabolic disorders that increase plasma P levels can cause anomalous mineralisation of soft tissues, as reported in patients that suffer from chronic kidney disease (CKD) [29,84] and in mice models of genetic diseases that cause hyperphosphatemia. Fgf23 is a hormone released by bone cells that down-regulates renal P reabsorption. Murine models with mutations of *Fgf23* or in genes encoding proteins involved in Fgf23 modifications, suffer from hypermineralisation adjacent to the growth plate in the primary spongiosa and hyperdense femur bones [85,86], similar to what observed in our HP zebrafish.

Pathological mineralisation that affects the vertebral column has been reported for zebrafish of the *enpp1* mutant strain. The lack of the ectonucleotide pyrophosphatase/phosphodiesterase-1 (Enpp1) reduces pyrophosphate, a mineralisation inhibitor generated by osteoblasts. The bones of juvenile *enpp1* mutants are hypermineralised and vertebral centra fuse [87], a phenotype similar to vertebral fusions in HP zebrafish. Notably, the increased frequency of vertebral body fusions in HP zebrafish is only diagnosed after two months. This suggests that the prolongation of the dietary treatment is required before an effect can be observed. Further studies are required to clarify the exact mechanisms of vertebral body fusion related to high dietary P content. 

Regarding the mechanical properties of the bone formed under the dietary treatment, a higher elastic modulus (increased material stiffness) was observed in the hypermineralised vertebral endplates of HP zebrafish compared to LP zebrafish. HP zebrafish also showed a tendency towards a higher elastic modulus compared to RP zebrafish. This suggests that not only a physiological increase in mineralisation, but also a dietary P-induced increase in mineralisation leads to a higher stiffness of bone [12,62,88]. It could be hypothesised that the increased stiffness of the vertebral centra likely increased the mechanical load on the intervertebral space while swimming, causing compression and tension of the intervertebral ligaments in the caudal region [89]. Tension is a well-known trigger for the mineralisation of ligaments and tendons [90,91]. Thus increased tension could trigger the mineralisation of the intervertebral ligaments, consequently leading to centra fusion [57].

### 3.4. Less Minerals but More Bone Production by Osteoblasts

Synchrotron X-ray tomographic microscopy allows the identification of mineralised and non-mineralised bone in the zebrafish vertebral bodies at a high resolution. The representative LP individual shows a vertebral body with an increased total bone volume in comparison to control and HP animals. The increase in bone volume is ascribed to a considerable increase of non-mineralised bone in the growth zone of the vertebral body endplates, neural and haemal arches and spines. Considering that animals of equal size were used from each group, it is intriguing to note that LP zebrafish show increased bone matrix formation. An increased production of collagen, consistent with the increased bone volume, suggests that osteoblasts are highly active at producing collagen matrix and this could explain the presence of enlarged endoplasmic reticulum cisternae in LP zebrafish [92,93]. The intensified matrix production does not influence collagen type I synthesis and post-translational modifications at the structural level. Collagen type I post-translational modification appears normal in LP zebrafish, as suggested by similar electrophoretic migration of α(I) chain bands in all dietary groups. Moreover, the progressive increase of collagen fibre diameter in the secreted bone matrix reflects normal fibrils maturation and aging [94]. Our observations agree with studies on Nile tilapia that show increased osteoid formation on pharyngeal bone in P-deprived animals [13]. Likewise, hypophosphatemic rats present increased osteoid width [14]. In mammals, osteoid undergoes several chemical modifications, designated as maturation, prior to mineralisation [95]. It has been suggested that increased osteoid production in P-depleted rats relates to a decreased rate of osteoid maturation, indicating a delay in the onset of mineralisation [14]. In Atlantic salmon, however, the non-mineralised bone formed under P-deficient conditions can mineralise completely if the animals receive a P-sufficient diet [12]. This, together with the normal post-translational modifications of collagen type I and the normal ultrastructure of collagen fibrils in LP zebrafish, argue in favour of normal bone matrix maturation also under low P conditions.

Maintaining bone mechanical stability could be a possible explanation for the increase of bone matrix production in LP zebrafish. As Ca and P contents in bone are always linked [10,12], the mechanical properties of bone change in accordance with the bones’ mineral content [62], as also shown in this study. LP zebrafish could increase collagen secretion to compensate for the lack of minerals and reduced stiffness. Osteocytes are mechanosensitive cells that regulate bone formation and bone resorption in response to mechanical load. Upon mechanical stimuli, osteocytes are activated and produce signalling molecules that increase the activity of osteoblasts [8]. In particular, prostaglandins secreted by osteocytes stimulate bone formation in response to mechanical load in vivo [96,97]. As the newly secreted matrix cannot mineralise due to insufficient P levels, stronger mechanical stimulation of osteocytes inside the soft non-mineralised bone could trigger an increased activity of the osteoblasts under LP conditions.

## 4. Materials and Methods 

### 4.1. Zebrafish and Ethical Statement

Wild type AB zebrafish were obtained from European Zebrafish Research Center (Eggenstein-Leopoldshafen, Germany). Zebrafish embryos were kept in petri dishes in fish water (1.2 mM NaHCO_3_, 0.01% instant ocean, 1.4 mM CaSO_4_, 0.0002% methylene blue) at 28 °C until 7 days post-fertilisation (dpf), then housed in ZebTEC semi-closed recirculation housing systems (Techniplast, Buguggiate, Italy) at 28 °C, pH 7.5 and conductivity 500 μS on a 14/10 light/dark cycle. Zebrafish from 7 to 21 dpf were fed three times a day alternating commercial dry food (ZM000, Zebrafish Management Ltd., Winchester, UK) and brine shrimp (Artemia cysts, Zebrafish Management Ltd., Winchester, UK). Fish were then fed for another week three times a day with the dry regular phosphorus (RP) diet (Table 3, see also the next section: experimental diets), until 28 dpf, to adjust them to this type of dry food. The nutrition trial started at 28 dpf: fish were randomly divided in three groups, grown in identical tanks with a density of 10 fish/L and fed three times a day with a low P (LP) diet, a regular P (RP) diet and a high P (HP) diet, respectively (Table 3). Samples were collected before the start of the experiment (T0 samples, 28 dpf) and after one and two months of dietary treatment (two and three months post-fertilisation, respectively). Fish were euthanised by tricaine (3-amino benzoic acidethylester) overdose (0.3%) and fixed for further analyses. The experiments were conducted in the centralised animal facility of the University of Pavia (Pavia, Italy). The experimental protocol was approved by the Italian Ministry of Health (Approval animal protocol No. 260/2020-PR, 26 March 2020).

### 4.2. Experimental Diets

Three experimental diets were formulated to have a total P content of 0.5%, 1.0% and 1.5%, termed LP diet, RP diet and HP diet, respectively (Table 3). P content for the control diet, RP, was based on the total dietary P requirement of 0.6–0.8% for different teleost species (Rainbow trout *Oncorhynchus mykiss*, Atlantic salmon *Salmo salar*, Pacific salmon *Oncorhynchus sp*., Carp *Cyprinus carpio*, European sea bass *Dicentrachus labrax*) [98]. LP and HP diets were formulated to have, respectively, a drastic reduction and an excess of total P content. Monoammonium phosphate (MAP) was used as dietary inorganic P supplement. MAP has a high P bioavailability and high P retention efficiency in teleost fish [71]. In order to keep all diets equal in nutrients, except for P concentration, MAP replaced the inert filler diatomaceous earth (Diamol, Imerys, Denmark). The experimental diets were formulated by SimplyFish AS (Stavanger, Norway, www.simplyfish.no) and produced by extrusion with subsequent crumbling to a suitable particle size by the Danish Technological Institute (Taastrup, Denmark, https://www.dti.dk). The P content of the product was verified at the University of Hohenheim (Stuttgart, Germany, https://www.uni-hohenheim.de) and determined with 5.04 g/kg diet, 9.84 g/kg diet and 14.64 g/kg diet for the LP, RP, and HP diet, respectively.

### 4.3. Morphometric Analysis

Fish were euthanised by tricaine overdose and lateral images were acquired with a M165FC stereomicroscope (Leica, Wetzlar, Germany) connected to a DFC425C digital camera (Leica, Wetzlar, Germany). The standard length (SL), described as the distance from the most anterior tip of the upper jaw to the most posterior region of the body where caudal fin rays insert [99], was measured using the ImageJ software (NIH, Bethesda, MD, USA) (T0 *n* = 96; one month treatment: LP *n* = 70, RP *n* = 34, HP *n* = 42; two months treatment: LP *n* = 59, RP *n* = 63, RP *n* = 47).

### 4.4. Whole Mount Staining with Alizarin Red S

Zebrafish of 28 dpf (*n* = 8), one month treated (LP *n* = 51, RP *n* = 21, HP *n* = 31) and two month treated (LP *n* = 41, RP *n* = 48, HP *n* = 32) fish were euthanised by tricaine overdose, fixed for 24 h in 4% paraformaldehyde (PFA) in 1× phosphate-buffered saline (PBS) at 4 °C and were stained according to the following protocol: 1.5% H_2_O_2_ in 0.25% KOH (2 h); distilled H_2_O (dH_2_O) (5 min); 0.01% Alizarin red S in 0.5% KOH (12 h); 1% KOH (2 h); 25% glycerol in 0.75% KOH (2 h); 50% glycerol in 0.5% KOH (2 h); 75% glycerol in 0.25% KOH (2 h); 100% glycerol (modified from [100]). Fish were analysed and imaged using an Axio Zoom V16 stereomicroscope (Carl Zeiss, Oberkochen, Germany) with oblique illumination equipped with a 5MP CCD camera. Lateral images of stained fish were used to quantitatively analyse mineralisation levels of vertebral endplates. The vertebral body in the transition region, described as the first caudal vertebra possessing elongated unfused haemal arches, drastically shortened ribs and absence of haemal spine [40], was considered for measuring the total vertebral length and the non-mineralised endplate length. Endplates represent the growth zone of vertebral centra, where the newly formed bone is deposited. Thus, endplates provide a valuable location to characterise bone formed during the dietary treatment. The non-mineralised endplate was expressed as a percentage of the total non-mineralised endplate length over the total vertebral length: (A + A’)/B (Figure 2c). Vertebral endplates with a non-mineralised percentage value greater than 10% were classified as low-mineralised, between 3% and 10% as intermediate mineralised, and less than 3% were considered fully mineralised. Fin rays were classified as low-mineralised if more than two segments were non-mineralised, intermediate mineralised if one or two segments were non-mineralised and fully mineralised if all segments were mineralised. Mineralisation levels of neural and haemal arches and dorsal and anal pterygiophores were qualitatively evaluated as low, intermediate or high depending on Alizarin red S distribution in the bone (Supplementary Figure S1).

### 4.5. Histological Analysis

Specimens for histological analysis were euthanised by tricaine overdose and fixed for 24 h in 2.5% PFA, 1.5% glutaraldehyde, 0.1 M sodium cacodylate buffer (pH 7.4) and 0.001% CaCl_2_ at 4 °C. Bone mineral detection was carried out on histological sections obtained from non-decalcified samples embedded in glycol methacrylate, according to [101]. Briefly, specimens were dehydrated in a graded series of acetone (30%, 50%, 70%, 90%, 100%) for 30 min each step. Samples were then impregnated with glycol methacrylate monomer solution (80 mL (2-hydroxyethyl)-methacrylate, 12 mL ethylene glycol monobuthyl ether, 270 mg benzoyl peroxide) for 60 min. For the second step of impregnation a fresh monomer solution was used for 24 h. For embedding 2% catalyst (1 mL *N*,*N*-dimethylaniline, 10 mL poly-ethylenglycole-200) was added to the monomer solution. Specimens were then embedded in polyethylene jars. Polymerisation took place at 4 °C for 48 h and was completed within another 24 h at room temperature. 3 μm sections were cut on a Microm HM 360 (Marshall Scientific, Hampton, NH, USA) automated microtome and were stained following the Von Kossa/Van Gieson staining protocol: 1% AgNO_3_ (45 min under UV light); dH_2_O (10 min, twice); 3% Na_2_S_2_O_3_ (5 min); dH_2_O (10 min, twice); Van Gieson counterstain (5 min); dH_2_O; air-drying and DPX mounting [102]. Images were acquired using an Axio Imager-Z1 microscope (Carl Zeiss, Oberkochen, Germany) equipped with an Axiocam 503 colour camera (Carl Zeiss, Oberkochen, Germany).

### 4.6. Transmission Electron Microscopy

Specimens treated for two months (*n* = 1 fish per dietary group) were euthanised by tricaine overdose and fixed for 24 h in 2.5% PFA, 1.5% glutaraldehyde, 0.1 M sodium cacodylate buffer (pH 7.4) and 0.001% CaCl_2_ at 4 °C. Fixed fish were decalcified in 0.1 M EDTA for 14 days. The decalcification solution was changed every 3 days. Specimens were subsequently rinsed in 0.1 M sodium cacodylate buffer with 10% saccharose and then post-fixed for 2 h in 1% OsO_4_ solution in 0.1 M cacodylate buffer containing 3% saccharose. After rinsing in buffer, samples were dehydrated in a series of graded ethanol solutions and embedded in epon epoxide medium [103]. Semi-thin 1 μm sections were cut on a Microm HM360 microtome (Marshall Scientific, Hampton, NH, USA), stained with toluidine blue at pH 9 for 2 min (0.5% toluidine blue, 1% Na_2_B_4_O_7_ in dH_2_O), rinsed with H_2_O and mounted with DPX. For transmission electron microscopy (TEM) analysis, ultrathin sections (about 70 nm) of the region of interest were prepared on an UltracutE ultramicrotome (Reichert-Jung, Buffalo, NY, USA), contrasted with uranyl acetate and lead citrate [104] and analysed with a Jeol JEM 1010 transmission electron microscope (Jeol Ltd., Tokyo, Japan) operating at 60 kV. Microphotographs were taken with a Veleta camera (Emsis, Muenster, Germany). TEM images were used to measure the diameter of collagen type I fibres in proximity of the osteoblasts (*n* = 300 per dietary group) and in the extracellular matrix (*n* = 300 per dietary group). Diameters were measured on fully transversely sectioned fibres using ImageJ software (NIH, Bethesda, MD, USA). Analysis of collagen fibres was based on previously established protocol [36], the number of fibres analysed exceeds the established one.

### 4.7. X-rays

X-rays of euthanised one month (LP *n* = 13, RP *n* = 8, HP *n* = 8) and two months (LP *n* = 16, RP *n* = 28, HP *n* = 22) treated zebrafish were acquired with a Faxitron Mx-20 (Faxitron, Tucson, AZ, USA) using 25 kV for 10 sec [36]. The Kodak DirectView Elite CR System and k-Pacs software (Kodak, Rochester, NY, USA) were used for image digitalisation.

### 4.8. Synchrotron X-ray Tomographic Microscopy

Fish treated with the experimental diets for two months (*n* = 1 fish per dietary group) were euthanised by tricaine overdose, fixed for 24h in 2.5% PFA, 1.5% glutaraldehyde, 0.1 M sodium cacodylate buffer (pH 7.4) and 0.001% CaCl_2_ at 4 °C and were dehydrated in a graded series of ethanol. Representative specimens of the dietary groups with similar size were selected by whole mount Alizarin red S staining of the abdominal region of the vertebral column. Synchrotron X-ray tomographic microscopy of the caudal region of the vertebral column [40] was performed at the TOMCAT beamline (X02DA) (Swiss Light Source (SLS), Paul Scherrer Institut (PSI), Villigen, Switzerland, www.psi.ch/sls). Scans were acquired with 1501 projections over 180˚ at 16 keV with an exposure time of 150 ms using a 10× objective resulting in an effective voxel size of 0.65 µm [105]. Radiographs were phase-retrieved using the Paganin algorithm, tomographically reconstructed and subsequently analysed using Amira 3.1.1 software (TermoFisher, Waltham, MA, USA). The mineralised parts of the vertebrae were identified applying a constant greyscale-threshold to all samples, the non-mineralised parts were manually selected on each projection. The resulting segmentations and virtual sections of the 10th caudal vertebral body [40] in LP, RP and HP fish were used to visualise the mineralised and non-mineralised parts of the vertebra, neural and haemal arches. Segmentations were also used to measure vertebral body parameters (length, height), arch and spine length, and the volumes of mineralised and non-mineralised parts of the vertebrae (Figure 5b).

### 4.9. Nanoindentation

Mechanical properties were assessed in the first four caudal vertebrae of 1 fish per dietary group according to previously established protocols [106]. Briefly, fish were embedded in PMMA, ground coplanar until the sagittal plane was exposed, and polished with 3 µm and 1 µm diamond suspension followed by final polishing with 0.05 µm aluminium-oxide suspension. Indentations were performed in depth-sensing continuous stiffness mode with a final depth of 300 nm. Indentations were placed in the proximal and distal vertebral body endplates and the central region of vertebrae (Figure 4a). The nanoindenter (Nano Indenter G200 equipped with a Berkovich diamond tip, Keysight Technologies, Santa Rosa, CA, USA) was calibrated on fused silica before and after each measurement. Based on the Oliver and Pharr method [107] and by applying a Poisson’s ratio of 0.3, the mechanical properties elastic modulus (E) and hardness (H) in the different vertebral regions were determined using in-house software (NanoSuite, Keysight Technologies, Santa Rosa, CA, USA).

### 4.10. Collagen Extraction from Bone

Bones were dissected following sacrifice from animals fed with the experimental P diets for two months (*n* = 2 fish per dietary group). Bones were defatted for 6 h in 0.1 N NaOH at 4 °C and then decalcified for 48 h in 0.5 M EDTA (pH 7.4) at 4 °C. The pepsin-soluble collagen fraction (PSC) was obtained by digesting tissues with 0.1 mg/mL pepsin in 0.5 M acetic acid at 4 °C for 48 h. The PSC was precipitated by 0.9 M NaCl in 0.5 M acetic acid overnight at 4 °C [108] and quantified using Sircol Soluble Collagen assay (Biocolor, Carrickfergus, UK). Equal amounts of collagen from each sample were loaded on 6% SDS-Urea-PAGE in non-reducing condition. Gels were stained overnight with 0.08 M picric acid, 0.04% Coomassie Brilliant Blue R250, rinsed in water and recorded with Versadoc3000 (Bio-Rad, Hercules, CA, USA).

### 4.11. Statistical Analysis

Quantitative variables are expressed as mean ± standard deviation, categories are expressed as percentages. Statistical comparison of the standard length values was based on the non-parametric Mann-Whitney test. Differences in the occurrence of skeletal malformations and in bone mineralisation levels were evaluated by means of Chi-squared test or the Fisher’s exact test. Statistical analysis was performed using Past 4.01 software [109]. Comparisons of mechanical properties was performed using ANOVA followed by a Bonferroni post hoc test. A *p*-value less than 0.05 was considered significant.

## 5. Conclusions

In this experiment, a new zebrafish model for low and high dietary P levels demonstrates that low P levels in the diet have no negative effect on bone matrix formation, although new bone matrix remains non-mineralised. Moreover, no vertebral centra malformations occur, indicating that other factors may trigger the development of skeletal deformities. In contrast, high dietary P levels lead to increased bone mineralisation, increased bone stiffness and fusion of vertebral centra. Neither the lack of mineralisation, nor the high mineralisation affect collagen post-translational modifications, as expected given that ER resident enzymes do not depend on P [45]. Increased production of normal collagen matrix is observed in animals from the LP group: organic matrix is continuously produced and collagen fibres mature despite the arrest of mineralisation. The current findings therefore support the idea that bone matrix secretion and bone mineralisation are uncoupled processes, and explain why non-mineralised bone produced under P deficiency conditions can mineralise completely when adequate dietary P is provided [12]. This new bone mineralisation zebrafish model can be used in biomedical research to obtain insights into bone mineralisation pathologies related to high and low mineralisation degree. The findings of this study may also benefit aquaculture research as a model for the effects of dietary P supply in farmed teleosts.

## Figures and Tables

**Table 1:** Standard length measures.

Groups		No. of Fish	Standard Length Mean ± SD (mm)	Pairwise *p*-Values		
				LP	RP	HP
28 dpf	T0	96	6.02 ± 0.84			
						
One Month Treatment	LP	70	8.90 ± 2.47		*p* = 0.001	*p* = 0.001
	RP	34	10.95 ± 3.13	*p* = 0.001		
	HP	42	10.76 ± 3.03	*p* = 0.001		
						
Two Months Treatment	LP	59	10.60 ± 2.79		*p* < 0.001	*p* < 0.001
	RP	63	12.16 ± 3.15	*p* < 0.001		*p* < 0.05
	HP	47	13.46 ± 3.62	*p* < 0.001	*p* < 0.05	

**Table 2:** Diagnosed vertebral body malformations.

Diets		No. of Fish	Vertebral Body Compression				Vertebral Body Fusion		
			No of Fish with Compression	Total No of Compression	Frequency		No. of Fish with Fusion	Total No. of Fusion	Frequency
One Month Treatment	LP	51	5	6	10%		4	4	8%
	RP	21	2	2	10%		0	0	0%
	HP	31	4	4	13%		1	1	3%
									
Two Months Treatment	LP	44	3	4	7%		2	2	5%
	RP	49	2	2	4%		7	10	14%
	HP	32	3	3	9%		9 *	18	28%

Vertebral centra with clear reduced anterior-posterior length were considered compressed. Statistical significance (* *p* = 0.006) was determined by pairwise Chi-squared test.

**Table 3:** Ingredients and chemical composition of the three experimental diets for zebrafish.

Ingredients (%)	Diets		
	LP	RP	HP
Rapeseed lecitin (Bergathin)	2.00	2.00	2.00
Krill meal	3.00	3.00	3.00
Wheat starch	18.77	18.77	18.77
Corn gluten meal	8.0	8.0	8.0
Wheat gluten meal	19.01	19.01	19.01
Soy protein concentrate	31.00	31.00	31.00
Capelin fish meal	5.00	5.00	5.00
Rapeseed oil	1.58	1.58	1.58
Peruvian fishoil	2.60	2.60	2.60
DL-Methionine	0.60	0.60	0.60
Biolys 54.6%	2.00	2.00	2.00
Lutavit C Aquastab 35%	0.10	0.10	0.10
Vitamin mix	0.50	0.50	0.50
Choline chloride 50%	1.50	1.50	1.50
Trace mineral mix (P free)	0.30	0.30	0.30
Monoammonium phosphate 26%	0.00	1.95	3.90
Diamol (diatomaceous earth)	4.00	2.05	0.10
Astaxanthin 10%	0.07	0.07	0.07
Total	100.00	100.00	100.00
			
**Chemical composition (g/kg)**			
Crude protein	497	508	520
Crude lipids	97	97	96
Crude ash	84	73	71
Calcium	4.84	4.79	4.65
Magnesium	1.96	1.98	1.99
Phosphorus	5.04	9.84	14.64

## Supplementary Materials

Can be found at https://www.mdpi.com/1422-0067/21/15/5429/s1. Table S1. Mineralisation levels of endoskeletal elements. Table S2. Mineralisation levels of dermal fin rays. Table S3. Synchrotron X-ray tomographic microscopy data analysis. Figure S1. Schematic representation of arches and pterygiophores mineralisation levels.
