# Supplementary material for: More Bone with Less Minerals? The Effects of Dietary Phosphorus on the Post-Cranial Skeleton in Zebrafish
Source: Int J Mol Sci. 2020 Jul 30;21(15):5429. doi: 10.3390/ijms21155429 (PMC7432380; doi:10.3390/ijms21155429)
Supplement: Supplementary file 1 [file ijms-21-05429-s001.pdf]

Table S1. Mineralisation levels of endoskeletal elements.

Endoskeletal element	Diets		N° of fish	Mineralisation level (Number of fish, %)			Pairwise p-values		
				Low	Intermediate	High	LP	RP	HP
Vertebral body endplates	one month treatment	LP	51	67%	25%	8%		p<0.001	p<0.001
		RP	21	19%	67%	14%	p<0.001		p<0.05
		HP	31	19%	35%	45%	p<0.001	p<0.05	
	two months treatment	LP	41	73%	27%	0%		p<0.001	p<0.001
		RP	48	23%	54%	23%	p<0.001		p<0.001
		HP	32	3%	31%	66%	p<0.001	p<0.001	
Neural and haemal arches	one month treatment	LP	51	25%	33%	41%		p<0.01	p<0.01
		RP	21	0%	24%	76%	p<0.01		
		HP	29	3%	24%	72%	p<0.01		
	two months treatment	LP	41	17%	39%	44%		p<0.001	p<0.001
		RP	48	0%	13%	88%	p<0.001		
		HP	32	0%	6%	94%	p<0.001		
Dorsal fin pterygiophores	one month treatment	LP	51	38%	31%	29%			p<0.01
		RP	21	14%	43%	43%			
		HP	29	14%	21%	66%	p<0.01		
	two months treatment	LP	41	51%	32%	17%		p<0.001	p<0.001
		RP	48	8%	42%	50%	p<0.001		
		HP	32	9%	19%	72%	p<0.001		
Anal fin pterygiophores	one month treatment	LP	51	29%	37%	33%			p<0.01
		RP	21	14%	38%	48%			
		HP	29	3%	31%	66%	p<0.01		
	two months treatment	LP	41	44%	34%	22%		p<0.001	p<0.001
		RP	48	6%	38%	56%	p<0.001		
		HP	32	9%	19%	72%	p<0.001		

Statistical significance was determined by Chi-squared test or the Fisher's exact test.

Table S2. Mineralisation levels of dermal fin rays.

Dermal skeletal element	Diets		N° of fish	Mineralisation level (Number of fish, %)			Pairwise p-values		
				**Low**	**Intermediate**	**High**	LP	RP	HP
Dorsal fin rays	one month treatment	LP	51	24%	41%	35%			p<0.05
		RP	21	10%	43%	48%			
		HP	31	7%	28%	66%	p<0.05		
	two months treatment	LP	41	46%	41%	12%		p<0.001	p<0.001
		RP	48	4%	27%	69%	p<0.001		
		HP	32	9%	19%	72%	p<0.001		
Anal fin rays	one month treatment	LP	51	27%	31%	41%			p<0.05
		RP	21	10%	29%	62%			
		HP	29	7%	28%	66%	p<0.05		
	two months treatment	LP	41	49%	34%	17%		p<0.001	p<0.001
		RP	48	4%	25%	71%	p<0.001		
		HP	32	9%	19%	72%	p<0.001		
Caudal fin rays	one month treatment	LP	51	22%	43%	35%		p<0.01	p=0.01
		RP	21	10%	14%	76%	p<0.01		
		HP	29	7%	24%	69%	p=0.01		
	two months treatment	LP	41	34%	41%	24%		p<0.001	p<0.001
		RP	48	0%	19%	81%	p<0.001		
		HP	32	3%	25%	72%	p<0.001		

Statistical significance was determined by Chi-squared test or the Fisher's exact test.

Table S3. Synchrotron X-ray tomographic microscopy data analysis.

Diets	Vertebral body measures (μm)		Arches-spine length (μm)		Bone volume (μm^3)		
	Length	Height	Neural arch-spine	Haemal arch-spine	Non-mineralised	Mineralised	Total
LP	261.26	192.88	461.18	437.11	18.3×10^5	10.1×10^5	28.4×10^5
RP	266.82	191.46	568.44	521.84	2.2×10^5	18.7×10^5	20.9×10^5
HP	259.41	171.97	723.57	615.64	0	13.8×10^5	13.8×10^5

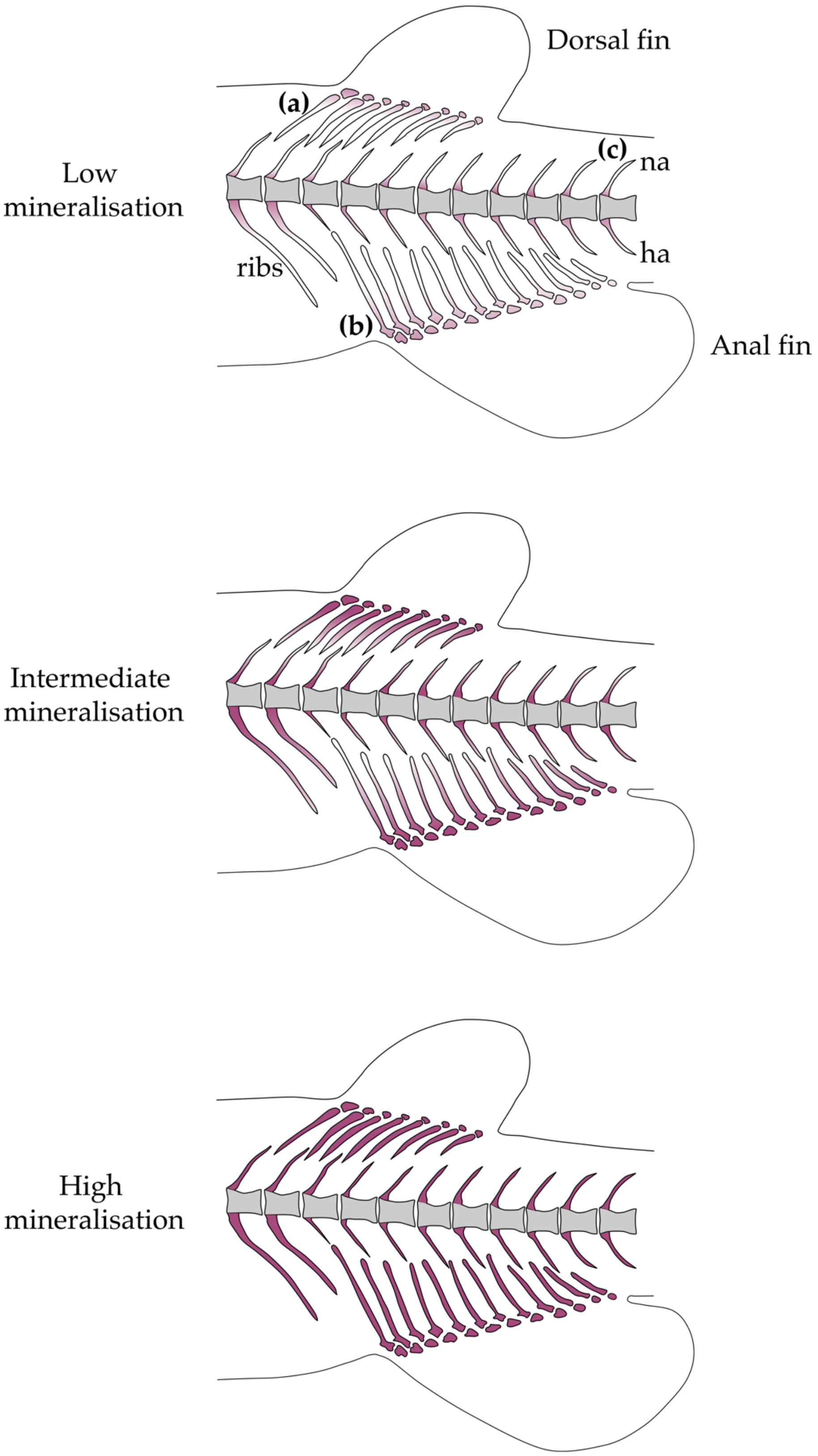

Figure S1. Schematic representation of arches and pterygiophores mineralisation levels. Mineralisation levels of dorsal **(a)** and anal **(b)** pterygiophores and neural (na) and haemal (ha) arches **(c)** were qualitatively evaluated as low, intermediate or high depending on Alizarin red S distribution in bone. Red: Alizarin red S staining; grey: vertebral centra.
